# Religion and culture

**DOI:** 10.1016/j.mpmed.2016.07.011

**Published:** 2016-10

**Authors:** Joshua Hordern

**Affiliations:** **Joshua Hordern MA PGDip MSt (Oxon) PhD (Edin)** is Associate Professor of Christian Ethics, Faculty of Theology and Religion, Healthcare Values Partnership, University of Oxford, UK. His research interests are compassion in healthcare, precision medicine and religion in public life. Competing interests: none declared

**Keywords:** Belief, communication, compassion, conscientious objection, culture, equality, religion

## Abstract

Religion, belief and culture should be recognized as potential sources of moral purpose and personal strength in healthcare, enhancing the welfare of both clinicians and patients amidst the experience of ill-health, healing, suffering and dying. Communication between doctors and patients and between healthcare staff should attend sensitively to the welfare benefits of religion, belief and culture. Doctors should respect personal religious and cultural commitments, taking account of their significance for treatment and care preferences. Good doctors understand their own beliefs and those of others. They hold that patient welfare is best served by understanding the importance of religion, belief and culture to patients and colleagues. The sensitive navigation of differences between people's religions, beliefs and cultures is part of doctors' civic obligations and in the UK should follow the guidance of the General Medical Council and Department of Health. In particular, apparent conflict between clinical judgement or normal practices and a patient's culture, religion and belief should be considered carefully. Doctors' own religion or culture may play an important role in promoting adherence to this good practice. In all matters, doctors' conduct should be governed by the law and arrangements for conscientious objection that are in effect.

**Key points**

• Religion, belief and culture are potential sources of moral purpose and personal strength for doctors and patients
• Personal beliefs and cultural practices are central to doctors' lives
• A doctor's own religion, culture or beliefs should not adversely affect their patients
• A patient's spiritual, social and cultural background is important to history-taking and handover
• A patient's beliefs may not be in line with their religion or culture's mainstream view
• A doctor's expression of their beliefs can be helpful in promoting patient care
• A patient's beliefs should not necessarily be decisive in determining their treatment
• Doctors should think carefully before articulating their own beliefs even if they are supported by law
• Religion or culture may play a positive role in promoting adherence to good, lawful practice
• Doctors with a conscientious objection may explain the reason for it, must not express disapproval and must inform patients of their rights to see another doctor
• Healthcare institutions provide vital environments for performing the civic obligation of democratic recognition and consideration of society's plural beliefs and views

## Recognizing the place of religion and culture in healthcare

Religion, belief and culture should be recognized in healthcare as potential sources of moral purpose and personal strength amidst the experience of ill-health, healing, suffering and dying. They should not be viewed solely or primarily as sources of problems in the delivery and reception of care. Rather, religion, belief and culture can mutually enhance the welfare of both clinicians and patients amidst the everyday challenges of patient experience and clinical practice. The conduct of medical practice should be informed by discerning application of this general principle. In particular, communication between doctors and patients and between healthcare staff should attend sensitively to the possible welfare benefits of religion, belief and culture.

The General Medical Council (GMC) specifically recognizes the importance of understanding spiritual, social and cultural factors when taking a history and of sharing relevant factors with colleagues when handing over (Good Medical Practice).^1^ The Department of Health for England and the Royal College of Psychiatrists emphasize the potential value of spirituality and prayer to patients' mental health and well-being. Moreover, the Department of Health affirms that ‘an individual's religion or beliefs are increasingly acknowledged as playing an important role in the overall healing process'.^2^

Equally, the GMC recognizes ‘that personal beliefs and cultural practices are central to the lives of doctors [and] that all doctors have personal values that affect their day-to-day practice’ and does not ‘wish to prevent doctors from practising in line with their beliefs and values’ where they are consistent with overall GMC guidance.^3^ With this in mind, a positive and open attitude to doctors' own religious and cultural beliefs is important for fostering a compassionate working environment. The general principle is that high-quality communication and ethics will be achieved by ‘medical professionals whose particular view of the world—of what is good and right, of what makes moral sense—forms them in the virtues that make them capable of practising medicine humanely’.^4^

## Fair and respectful treatment

In short, healthcare institutions are an important context in which people's personal religious and cultural commitments must be recognized as worthy of democratic respect and dignity. This recognition is limited in two ways. First, recognition should not give rise to any unlawful action. Second, recognition does not entail the approval or endorsement of any particular belief. The GMC emphasizes the obligation on doctors to ‘treat patients fairly and with respect whatever their life choices and beliefs’.^1^ This means that no patient should be disadvantaged because of their beliefs, but equally it does not mean that their beliefs should necessarily be decisive in determining their treatment. This is especially important where there is an apparent conflict between clinically indicated recommendations and a patient's religious or cultural commitment.

For example, an individual's interpretation of life and health may entail that suffering is not to be eliminated but rather endured and alleviated where possible. This view allows that suffering can be a time of learning and disclosure, even redemption and reconciliation. ‘What it is for a person to suffer or to feel compassion is contextualized, often within … traditions’ of morality, religion and culture.^5^ By way of illustration, some Buddhist thought emphasizes maintaining consciousness in pain. This emphasis would have a practical impact on decisions about the choice and appropriateness of pain relief measures. Similarly, for many religions, life does not end in death. Such belief is worthy of recognition and gives rise to treatment and care preferences that are relevant to a judgement of what is in the best interests of the patient.^2^

## Understanding sensitivities

Apparent conflict between clinical judgement and culture, religion and belief should be approached sensitively and without assumptions about the significance of the belief to the patient's attitudes and preferences. An individual's beliefs may not be wholly in line with their religion or culture's normative teaching. Therefore doctors should be sensitive not only to the strength of a patient's belief, but also to the particular interpretation of religion or culture the individual holds.^2^ Paying attention to the nature of cultural or spiritual factors in taking a patient history therefore requires subtlety and attention. An open question such as ‘Do you have a faith or belief that helps you at difficult times?’ may provide the opportunity for patients to articulate their wishes and religious understanding. Listening carefully to the answer to such a question will help to avoid any assumptions being made that might adversely affect the patient's care.

In particular, a doctor's own religion, culture or beliefs should not adversely affect patients,^2^ either in the interpretation of a patient's religion or culture, or in the expression of the doctor's own beliefs. The GMC advises that ‘You must not express your personal beliefs (including political, religious and moral beliefs) to patients in ways that exploit their vulnerability or are likely to cause them distress’.^3^ This does not imply that a doctor may not express their own beliefs, but rather it forbids them doing so exploitatively or in ways likely to be distressing. There are commonly circumstances where a doctor's expression of their beliefs is appropriate in promoting patient care. For example, a doctor's personal understanding and experience of Hindu or Muslim rites can provide reassurance to patients or relatives concerned about following prescribed mourning or burial practices.^2^

Doctors should, however, think carefully before articulating their own beliefs even if they are supported by law. For example, a belief that brainstem death is actual death is in line with UK law. However, the articulation of such a belief by a doctor, especially in circumstances where organ donation is a factor, may be experienced as hostile by patients or their relatives, such as some Buddhists and Christians, who believe that only cardiorespiratory death is actual death^3^; this is also discussed by David Jones – see Further reading.

Similarly, a doctor may have a philosophical belief, again in line with UK law, that a pre-sentient fetus, especially one severely disabled and not compatible with life outside the womb, is not a child. But this belief should not adversely affect and cause distress to patients who may either be uncertain about or profoundly disagree with such a philosophical belief.^2^ For example, many Christians, such as those whose views are represented by the Society for the Protection of Unborn Children, consider such a belief wrong and thus an inappropriate basis for care.

In many circumstances, it is difficult to know whether adverse effects will occur if doctors express their views. Much turns on the manner in which such matters arise and are discussed. Good doctors will have an awareness of their own commitments and an understanding of the beliefs and commitments of others. They will also believe that patient welfare is best served by taking seriously the possibility that religion, belief and culture may be important factors in patients' and colleagues' lives.

## Legal obligations

In all matters, doctors' conduct should be governed by the legal regime in operation in their working context. UK equality legislation provides that services should be provided without discrimination based on protected characteristics (The UK Equality Act 2010 lists the following protected characteristics: age, disability, gender reassignment, race, marriage and civil partnership, pregnancy or maternity, religion or belief, sex and sexual orientation). For example, a religious belief that a particular sexual lifestyle or the use of alcohol is wrong should not adversely affect patients' care. Such beliefs are themselves worthy of respect and protection in a plural, democratic society, are not unlawful and may be fully compatible with an affirmation of human dignity. Nonetheless, the GMC emphasizes that ‘You must not refuse or delay treatment because you believe that a patient's actions or lifestyle have contributed to their condition.’^1^ Even if this is a doctor's deeply held belief, it should not translate into any implication of or expression of condemnation.

Religion or culture can itself play an important role in promoting adherence to such good practice. For example, a Christian or other well-grounded commitment to the importance of mercy in human life can underpin some doctors' commitment to treat the health consequences of patients' damaging lifestyle choices without any condemnatory attitude towards the patient.^3^ Such an attitude in no way suggests that treatment and recommendations should avoid providing information and advice so that a patient may decide to change their lifestyle and avoid actions deleterious to their future health.^1^

There are occasions when some interpretations of religion and cultural traditions may lead to unlawful actions such as carrying out or assisting in female genital mutilation. In such cases and depending on the circumstances, there are mandatory reporting and safeguarding procedures that doctors must carry out, as specified, in England, by the Department of Health.

## Conscientious objection

The circumstances in which conscientious objection is available vary across legal jurisdictions. The GMC advises that any ‘conscientious objection must not imply/express disapproval although you may mention the reason’ for that conscientious objection. Doctors are therefore permitted to explain the reason for not carrying out a procedure but should do so bearing in mind the concerns about sensitivity discussed above.

Doctors who have a conscientious objections ‘must tell [patients] about their right to see another doctor and make sure they have enough information to exercise that right … If it is not practical for a patient to arrange to see another doctor, [they] must make sure that arrangements are made for another suitably qualified colleague to take over [their] role’.^1^, ^3^ The act of making such arrangements is itself morally complicated and difficult to describe in universally agreed terms. Some would see it as involving complicity in a moral wrong, while others, who similarly hold, for example, abortion to be a wrong, would see making arrangements for another colleague to take over as reasonable.

Common circumstances where a conscientious objection is acted upon currently include abortion, fertility treatment and the withdrawal of life-prolonging treatment from patients who lack capacity. If physician-assisted suicide or euthanasia ever became legal in any part of the UK, the same provision for conscientious objection would seem appropriate. But any doctor who currently assists a suicide or performs an act of euthanasia, perhaps even citing a positive claim on their conscience to do so, would be acting illegally under UK law.

## Democratic recognition as civic obligation

In communication and ethical discernment about religion and culture, doctors should seek to understand patients' and colleagues' beliefs, be sensitive to them in practice and comply with the law. It is a general principle that everyone deserves careful recognition and consideration of their beliefs and views in a democratic society. Healthcare institutions are vital environments for the realization of this principle in practice. Doctors should ask sensitively, gain information relevant to the care of patients and contribute where appropriate. In this way, doctors have a civic obligation to enhance a society's overall quality of understanding and sensitivity to the plural religious and cultural views that characterize its life.
